# Metabolic Reprogramming in Health and Disease

**DOI:** 10.3390/ijms21082768

**Published:** 2020-04-16

**Authors:** Grazia Chiellini

**Affiliations:** Department of Pathology, University of Pisa, 56100 Pisa, Italy; grazia.chiellini@unipi.it

## Abstract

This editorial aims to summarize the six scientific papers that contributed to this Special Issue.

Metabolism is broadly defined as the sum of biochemical processes in living organisms that either produce or consume energy. It is widely acknowledged that metabolic perturbations—often genetically programmed—accompany common human diseases. Among these, cancer is a prime example of a disease with genetically-defined, pathological metabolic perturbations. Indeed, metabolic reprogramming, such as enhanced aerobic glycolysis, mutations in the tricarboxylic acid (TCA) cycle metabolic enzymes, and dependence on lipid and glutamine metabolism are key characteristics of cancer cells. Therefore, addressing such metabolic perturbations is a very promising strategy for anti-cancer therapies that, in combination with conventional chemotherapeutic agents, may enhance therapeutic efficacy and clinical outcomes.

The understanding that metabolic changes occurring in cancer cells may have an impact on immune cell functionality, and contribute to tumor immune evasion, has fueled a growing interest in developing techniques that target metabolism for immunotherapy [1]. As elegantly reviewed in this Special Issue by Cassim and Pouyssegur over the last two decades, there has been increasing knowledge that tumor microenvironment metabolically modulates immune responsiveness, ultimately leading to re-consider how targeting tumor metabolism may hold new promising therapeutic approaches [2].

Alterations in mitochondrial function have long been considered a hallmark of cancer [3]. Multiple studies reported differences in the mitochondrial membrane lipid composition of cancer cells as compared to non-cancer cells, raising the question about the impact of these modifications for tumor biology and survival. In this light, the study of Bellance et al. demonstrates that Doxorubicin (DXR), a drug widely used in chemotherapy, also known as Adriamycin, alters the mitochondrial membrane composition associated with bioenergetic impairment and cell death in human cancer cells [4]. The remodeling of the mitochondrial membrane was explained by phosphatidylserine decarboxylase (PSD) inhibition by DXR. PSD catalyzes phosphatidylethanolamine (PE) synthesis from phosphatidylserine (PS), and DXR altered the PS/PE ratio in the mitochondrial membrane. Therefore, the authors provide the novel insight that the sensitivity of DXR treatment in chemotherapy is strongly related to the cell capacity for biosynthesis of PE.

Many nuclear oncogenes or tumor suppressors play a direct role in regulating transcription, and nearly all such proteins are modified by O-GlcNAcylation, namely the attachment of O-linked N-acetylglucosamine (O-GlcNAc) moieties to the protein [5]. A single pair of enzymes—O-GlcNAc transferase (OGT) and O-GlcNAcase (OGA)—control the dynamic cycling of this protein modification in a nutrient- and stress-responsive manner. The serine/threonine hydroxyl-linked N-Acetylglucosamine (O-GlcNAc) transferase (OGT) has been shown to drive pulmonary arterial smooth muscle cell (PASMC) proliferation in idiopathic pulmonary arterial hypertension (IPAH), a vasculopathy characterized by elevated pulmonary vascular resistance due to vasoconstriction and/or lung remodeling, such as plexiform lesions, as well as cell proliferation and vascular and angiogenic dysfunction [6]. The study of Barnes et al. provides for the first time clear evidence of a role for the OGT/O-GlcNAc axis in modulating vascular endothelial growth factor (VEGF) expression and vascularization in IPAH [6]. Their findings provide greater insight into the potential role that altered glucose uptake and metabolism may have on the angiogenic process and the development of plexiform lesions, in turn suggesting that OGT/O-GlcNAc axis may be a potential therapeutic target for treating the angiogenic dysregulation that is present in IPAH. 

It is widely known that mitochondrial dysfunction and oxidative stress are associated with the development of neurodegenerative diseases. Among them, amyotrophic lateral sclerosis (ALS) is an incurable progressive neurodegenerative disease that affects nerve cells in the brain and spinal cord, causing loss of muscle control. ALS reduces muscle strength, modulates muscle metabolism through lowering citrate synthase (CS) and increasing cytochrome c oxidase and malate dehydrogenase activities, and elevates oxidative stress markers in skeletal muscle [7]. Therefore, metabolic reprogramming in skeletal muscles in the human and animal models of ALS may be an important factor in the progression of the disease. In their study, Flis et al. hypothesized that swim training, a modulator of cellular metabolism via changes in muscle bioenergetics and oxidative stress, ameliorates the reduction in muscle strength in ALS mice [7]. Indeed, they clearly showed that swim training significantly decreased the decline in muscle strength in ALS animals, and provides, for the first time, compelling evidence that only citrate synthase (CS) activity is correlated with changes in muscle strength in ASL animals with first symptoms of the disease.

The melanocortin 4 receptor (MC4R) is a key player in hypothalamic weight regulation and energy expenditure as part of the leptin–melanocortin pathway [8]. Mutations in this G protein coupled receptor (GPCR) are the most common cause for monogenetic obesity, which appears to be mediated by changes in the anorectic action of MC4R via G_S_-dependent cyclic adenosine-monophosphate (cAMP) signaling, as well as other signaling pathways [8]. To study the potential bias in the effects of MC4R mutations between the different signaling pathways, Paisdzior et al. extensively investigated three major MC4R mutations: a Gs loss-of-function (S127L), a Gs gain-of-function mutant (H158R), as well as the most common European single nucleotide polymorphism (V103I) [9]. Overall, their data point out the complexity of MC4R signaling and its dependence on specific ligands. This implies that a cautious and standardized functional characterization is necessary to evaluate the functional impact of different MC4R mutations. This observation is of great importance, as there is now growing evidence that more MC4R pathways are relevant for the development of obesity than previously expected. 

The crucial role played by thyroid hormone (TH) on energy expenditure and body weight in humans is well recognized. Hyperthyroidism is characterized by increased resting energy expenditure, weight loss, and heat intolerance. On the other hand, reduced resting energy expenditure, weight gain, and cold intolerance are hallmarks of hypothyroidism. The action of metabolically active TH metabolites involves a wide variety of stimuli and conditions that have still not been completely unraveled. Rutigliano et al. elegantly review the relevance of 3-iodothyronamine (T1AM) in the regulation of energy homeostasis and metabolism, along with its potential therapeutic applications in metabolic disturbances [10]. As highlighted by the authors, the availability of powerful methods of investigation in the field of metabolomics allowed to directly demonstrate that the chronic treatment of mice with pharmacologic doses of T1AM rapidly induces lipid mobilization, subsequently shifting the fuel source from carbohydrates to lipids, and limiting lipogenesis. Notably, the molecular targets responsible for T1AM metabolic reprogramming activity appear to relay on independent events initiated either at receptors on the plasma membrane, such as TAAR1 and ADRA2A, or intracellularly upon T1AM uptake, including mitochondrial F0F1-ATP synthase. In light of this, T1AM appears to be endowed with pleiotropic functions that may confer to this endogenous TH metabolite, as well as to recently developed synthetic mimickers, such as SG-2 [11], great promise as novel pharmacological agents for the prevention and/or treatment of excessive weight and obesity, which are the major determinants of metabolic syndrome, one of the major causes of preventable premature death worldwide. 

In summary, all articles published in this Special Issue thoroughly describe the most interesting current directions in metabolic reprogramming studies. Cellular metabolic reprogramming is an important mechanism by which cells rewire their metabolism to promote proliferation and cell growth. This process has been mostly studied in the context of pathological processes, with a special focus on cancer, while little is known about its relevance for non-pathological conditions and how it affects animal physiology as a whole. It is a pleasure for the Guest Editor to thank all the authors for their important contributions to this Special Issue and the advancement of knowledge in future studies.
